# Infodemiological data of Ironman Triathlon in the study period 2004–2013

**DOI:** 10.1016/j.dib.2016.08.040

**Published:** 2016-08-27

**Authors:** Sofiane Mnadla, Nicola Luigi Bragazzi, Mehdi Rouissi, Anis Chaalali, Anna Siri, Johnny Padulo, Luca Paolo Ardigò, Francesco Brigo, Karim Chamari, Beat Knechtle

**Affiliations:** a Faculty of Humanities and Social Sciences, Tunis University, Tunisia; b School of Public Health, Department of Health Sciences (DISSAL), Genoa University, Genoa, Italy; c Department of Neuroscience, Rehabilitation, Ophthalmology, Genetics, Maternal and Child Health (DINOGMI), Section of Psychiatry, Genoa University, Genoa, Italy; d Department of Mathematics (DIMA), University of Genoa, Genoa, Italy; e Tunisian Research Laboratory ‘‘Sport Performance Optimisation’’, National Center of Medicine and Science in Sports, Tunis, Tunisia; f University eCampus, Novedrate, Italy; g Faculty of kinesiology, University of Split, Split, Croatia; h Department of Neurological, Biomedical and Movement Sciences, School of Exercise and Sport Science, University of Verona, Verona, Italy; i Department of Neurology, Franz Tappeiner Hospital, Merano, Italy; j Department of Neurological, Biomedical, and Movement Sciences, Section of Neurology, University of Verona, Verona, Italy; k Athlete and Health Performance Research Center, Aspetar, Doha, Qatar; l Institute of General Practice and for Health Services Research, University of Zurich, Zurich, Switzerland; m Gesundheitszentrum St. Gallen, St. Gallen, Switzerland

**Keywords:** Digital era, Google Trends, Infodemiology, Ironman Triathlon, Web 2.0

## Abstract

This article reports data concerning the Internet-related activities and interest for Ironman Triathlon competition. Google Trends (GT) was used and mined from 2004 onwards. The interest for Ironman Triathlon was found to be cyclic over time. The Triathlon-related Internet activities negatively correlated with the number of finishers per year (Pearson׳s correlation *r*=−0.690, *p*-value<0.05), while an increasing participation of female athletes who were less likely to surf the Internet could be noticed (*r*=−0.811, *p*-value<0.05). Further, younger athletes, who were more likely to access the web, were underrepresented in the Ironman Triathlon event. Moreover, there was a correlation between the biking time and the Internet query volumes (*r*=0.590, *p*-value<0.05), and, in particular, for the male athletes (*r*=0.664, *p*-value<0.05). Finally, the countries which most contributed to the Internet query volumes were those with the highest number of medals.

**Specifications Table**

**Table:** 

Subject area	*Sports sciences*
More specific subject area	*Sports data mining*
Type of data	*Graphs, heat-maps*
How data was acquired	*Outsourcing of Google Trends site and the Ironman site*
Data format	*Raw and Analyzed*
Experimental factors	*Google Trends search volumes were obtained through graphs and heat-maps*
Experimental features	*Validation of Google Trends-based data with “real-world” data taken from the Ironman site was performed by means of correlational analysis*
Data source location	**Worldwide**
Data accessibility	Data are within this article

**Value of the data**

• Google Trends (GT)-based data (*infodemiological* data) could be useful for scientific community and researchers in that they show good correlation with “real world” data obtained from the Ironman site, thus proving to be reliable.
• These data could be further statistically processed, analyzed, refined and validated.
• These data could be used to understand sports-related web activities.

## 1 Data

**Fig. 1:**
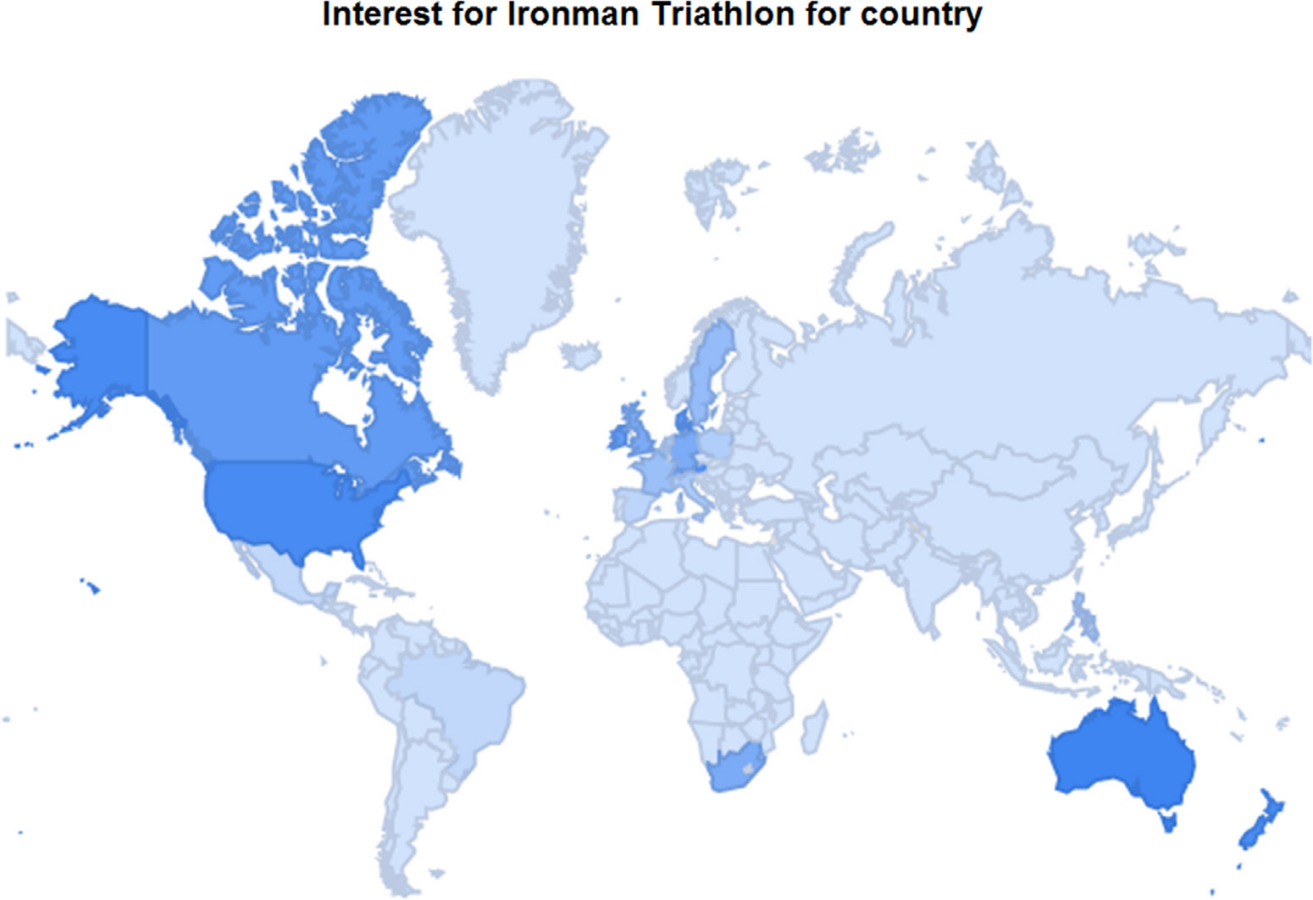
Heat-map of interest for Ironman Triathlon for each country.

**Fig. 2:**
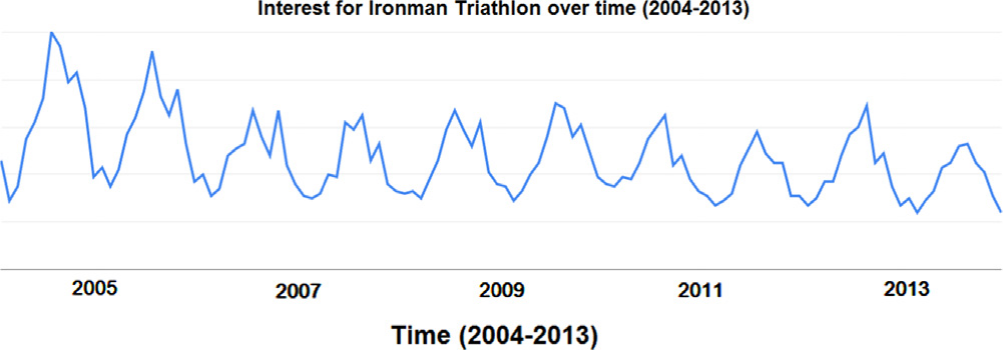
Interest for Ironman Triathlon over time in the period 2004–2013, worldwide.

**Fig. 3:**
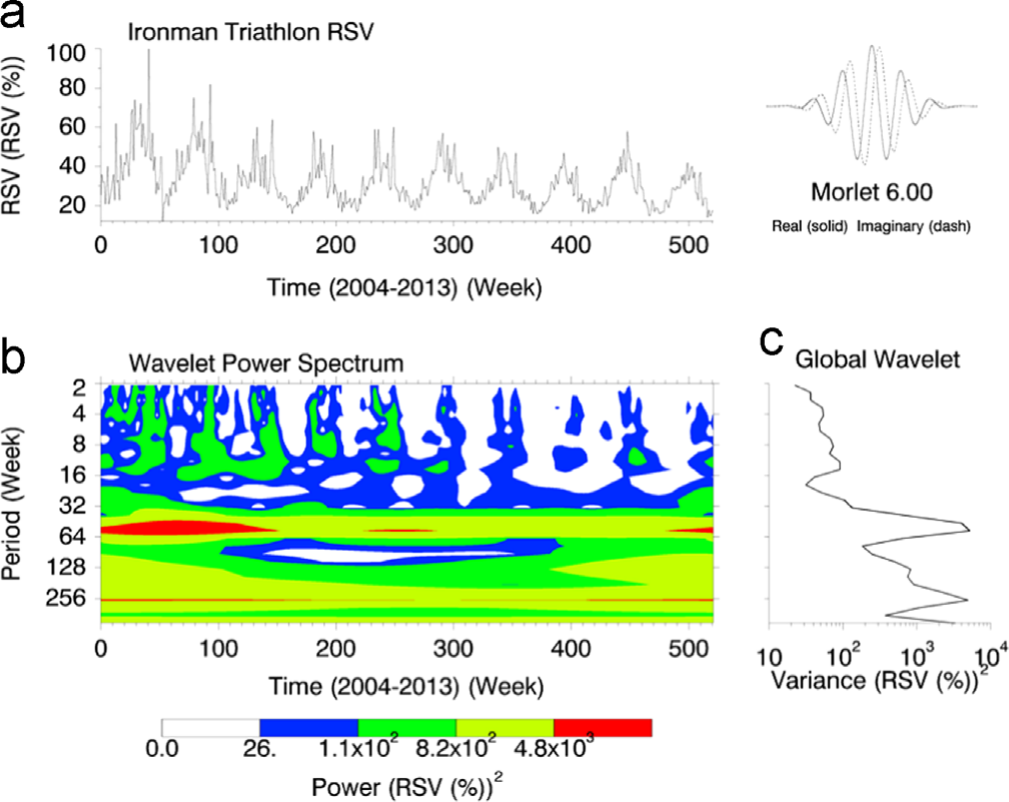
Wavelet Spectral Analysis of Ironman Triathlon-related web searches.

**Fig. 4:**
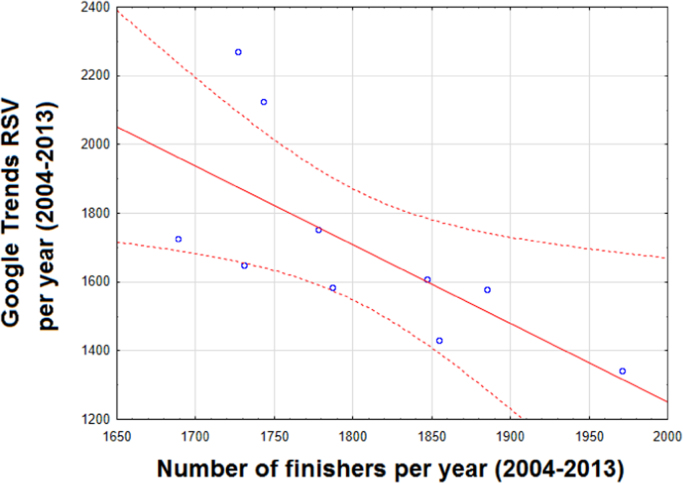
Correlation between Ironman Triathlon-related web activities and number of finishers per year.

**Fig. 5:**
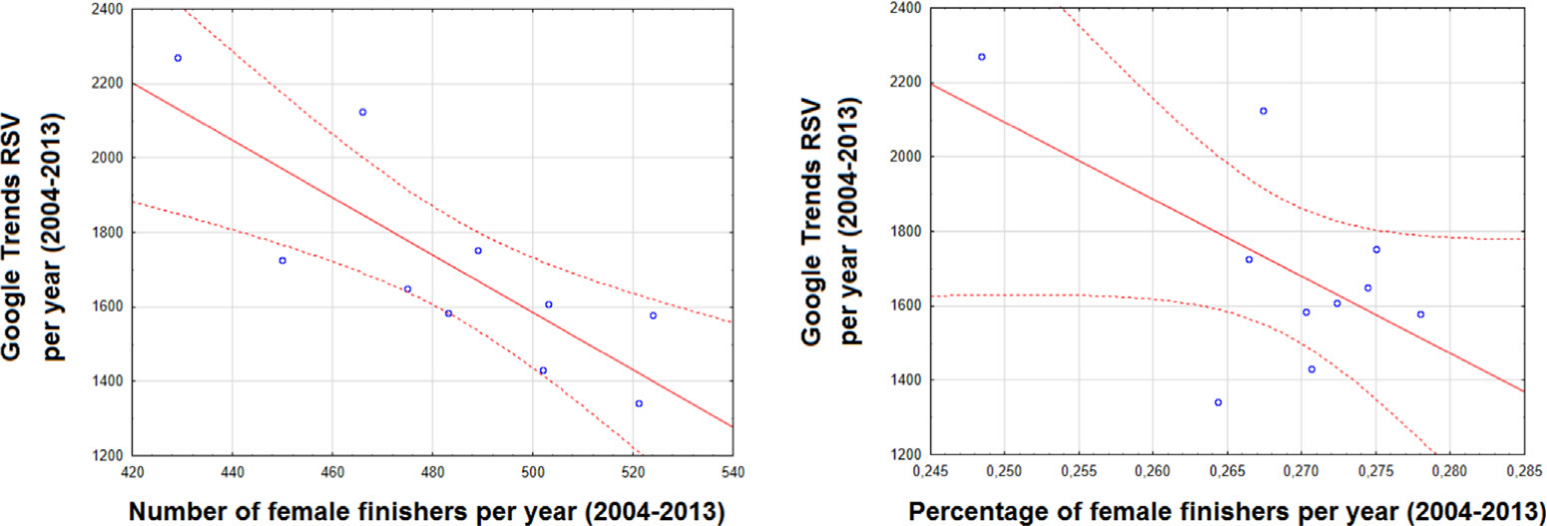
Correlation between Ironman Triathlon-related web activities and number/percentage of female finishers per year.

**Fig. 6:**
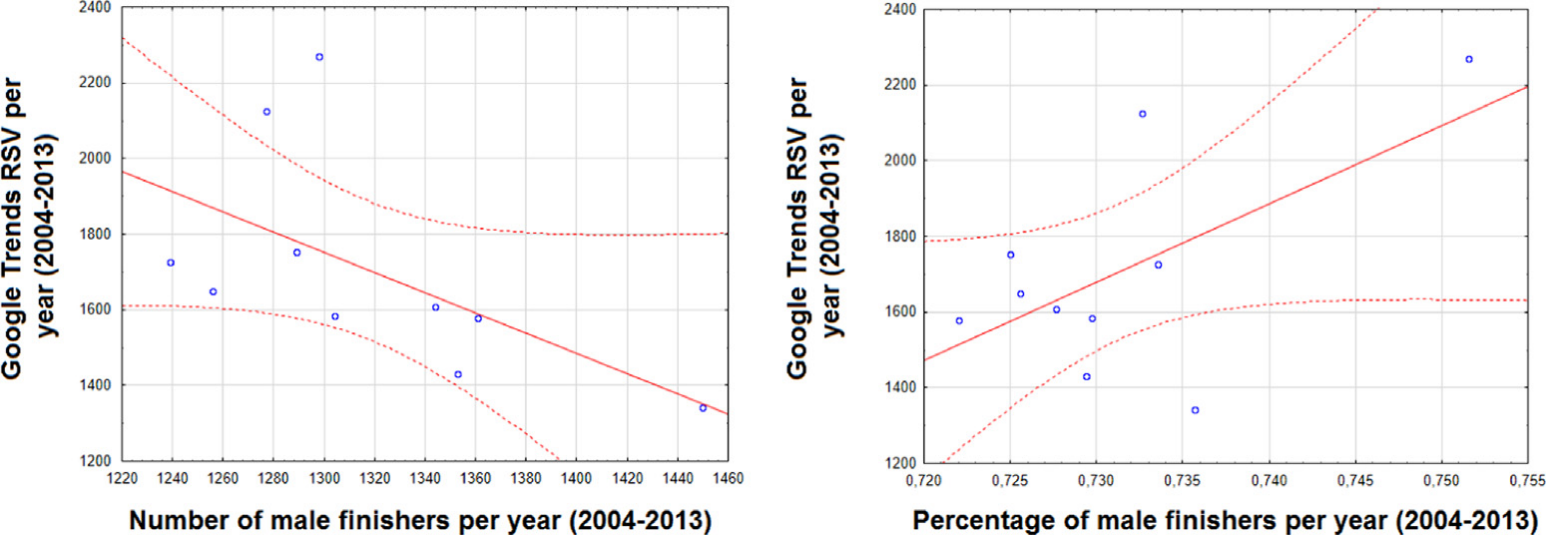
Correlation between Ironman Triathlon-related web activities and number/percentage of male finishers per year.

**Fig. 7:**
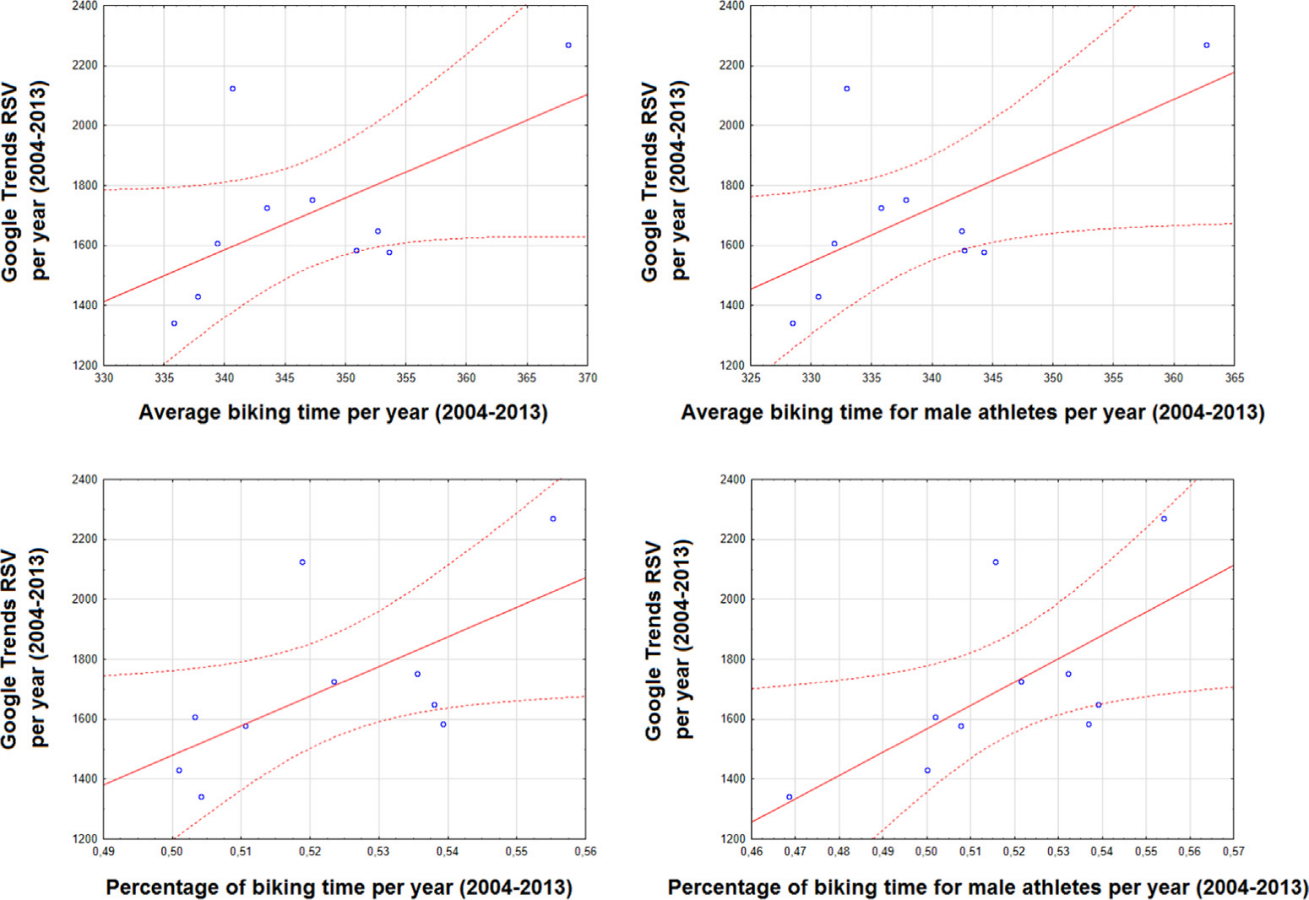
Correlation between Ironman Triathlon-related web activities and average biking time per year/percentage of biking time per year (overall and for male athletes).

This article contains infodemiological data on Ironman Triathlon searched worldwide in the study period 2004–2013, obtained from Google Trends (GT) (Fig. 1, Fig. 2). These data showed a cyclic pattern (Fig. 3) and well correlated with “real-world” data obtained from the Ironman Triathlon site for the same study period (Fig. 4, Fig. 5, Fig. 6, Fig. 7).

## 2 Experimental design, materials and methods

GT (freely available at https://www.google.com/trends) was used to explore Internet activities and interest related to Ironman Triathlon competition [1]. GT was searched worldwide, looking for “Ironman triathlon” as keyword, and using “search topic” as search strategy option, from its inception until 2013. “Real-world” statistical data were collected from the Ironman Triathlon site (available at http://ironmanworldchampionship.com) for the same study period 2004–2013.

In order to capture regular time patterns, spectral analysis was carried out using algorithms written in Matlab, freely accessible at http://paos.colorado.edu/research/wavelets/ [2].

Correlational analysis was carried out between the GT-based search volumes and the “real-world” statistical data about Ironman Triathlon. All statistical analyses were performed using commercial software, namely the Statistical Package for Social Science version 23.0 (SPSS, IBM, IL, USA) and STATISTICA version 12 (StatSoft Inc., Tulsa, OK, USA). Figures with a *p*-value<0.05 were considered statistically significant.

## Conflicts of interest

The authors declare no conflicts of interest.

## References

[1] Knechtle B., Nikolaidis P.T., Rosemann T., Rüst C.A. (2016). Ironman Triathlon. Prax. (Bern. 1994).

[2] Torrence C., Compo G.P. (1998). A practical guide to wavelet analysis. Bull. Am. Meteorol. Soc..

